# Loss of superhydrophobicity of hydrophobic micro/nano structures during condensation

**DOI:** 10.1038/srep09901

**Published:** 2015-04-23

**Authors:** HangJin Jo, Kyung Won Hwang, DongHyun Kim, Moriyama Kiyofumi, Hyun Sun Park, Moo Hwan Kim, Ho Seon Ahn

**Affiliations:** 1 Division of Advanced Nuclear Engineering, POSTECH, Pohang, Gyungbuk, Republic of Korea; 2 Department of Mechanical Engineering, POSTECH, Pohang, Gyungbuk, Republic of Korea; 3 Division of Mechanical System Engineering, Incheon National University, Incheon, Republic of Korea

## Abstract

Condensed liquid behavior on hydrophobic micro/nano-structured surfaces is a subject with multiple practical applications, but remains poorly understood. In particular, the loss of superhydrophobicity of hydrophobic micro/nanostructures during condensation, even when the same surface shows water-repellant characteristics when exposed to air, requires intensive investigation to improve and apply our understanding of the fundamental physics of condensation. Here, we postulate the criterion required for condensation to form from inside the surface structures by examining the grand potentials of a condensation system, including the properties of the condensed liquid and the conditions required for condensation. The results imply that the same hydrophobic micro/nano-structured surface could exhibit different liquid droplet behavior depending on the conditions. Our findings are supported by the observed phenomena: the initiation of a condensed droplet from inside a hydrophobic cavity, the apparent wetted state changes, and the presence of sticky condensed droplets on the hydrophobic micro/nano-structured surface.

Superhydrophobic surfaces have been investigated widely due to their useful properties that include self-repelling^1^,^2^, anti-sticking^3^, anti-fouling^4^ and self-cleaning^5^,^6^,^7^,^8^,^9^,^10^ characteristics. These are important for condensation heat transfer^2^,^11^,^12^,^13^,^14^, anti-frost coating on windows^15^, anti-fouling paints^16^,^17^, anti-ice adhesion coatings^18^,^19^, reducing the frictional pressure drop^20^ and cleaning surfaces^21^,^22^,^23^. However, superhydrophobic surfaces lose their useful characteristics at high supersaturated water vapor conditions (*P_vapor_/P_sat_*, *S* where *P_sat_ = P_sat_*(*T_surface_*)) although they have superhydrophobic properties under atmospheric conditions^24^,^25^. Researchers have reported that condensed water fills the surface structures, and that the wetted structures are responsible for the loss of superhydrophobicity^2^,^26^,^27^,^28^,^29^. A droplet was stickier on the surface filled with liquid rather than a hydrophobic smooth surface because it filled with liquid^24^,^25^. To date, studies of the loss of surface superhydrophobicity have focused on the effect of structured surface morphology on the state of droplet^26^,^30^,^31^,^32^, but the conditions surrounding the condensation of droplets have not been theoretically investigated. Here, we describe a theoretical analysis of the mechanism by which a superhydrophobic surface loses its novel characteristics at high supersaturation conditions, supported with experimental evidence. The initiation of condensation from inside a hydrophobic cavity was experimentally observed using environmental scanning electron microscopy (ESEM). A theoretical size criterion for determining whether condensation occurs inside a hydrophobic cavity or not is proposed by comparing the grand potentials of the system. Our analysis of droplet formation from inside the hydrophobic nano-interstice is supported by changes in the observed apparent wetted state as the number of condensation nucleation sites are varied, and by the loss of superhydrophobicity on a hydrophobic micro/nano-structured (HMN) surface. This study provides a fundamental understanding of the wetted state of condensed droplets on HMN surfaces, and will contribute to many relevant multi-phase systems and self-cleaning applications.

The structured surfaces were obtained by an anodic oxidation method^33^. Zirconium alloy plates, 40 × 30 mm, were used for the samples. The conductivity of the plates was similar to stainless steel. To gain hydrophobicity, the samples were coated by poly tetra fluoro ethylene (PTFE) using a spin coater (Fig. 1). The characteristics of the surface structures were observed by high-resolution field-emission scanning electron microscopy (FE-SEM) with focused ion beam (FIB), as shown in Fig. 1. The microscale structures were composed of nanoscale grains. The diameter of the nano-grains was ranged from 22 to 49 nm (Supplementary Information, Section 10). Additionally, we prepared smooth zirconium alloy plates coated with PTFE to provide a hydrophobic smooth (HS) surface for comparison (Supplementary Information, Section S6). The coating thickness of the PTFE, measured by a surface profiler, was 94 nm, and the uniformity of the coating was confirmed (Supplementary Information, Sections S7 and S9). The contact angle of a droplet deposited on a specimen was ~ 166° for the HMN surface and ~ 112° for the HS surface in atmospheric air.

The fundamental behaviors of the condensed liquid droplets were characterized by ESEM^2^,^12^,^13^,^14^,^26^,^31^,^32^,^34^. In ESEM imaging process, large viewing area and small potential were chosen for minimizing the undesirable effects due to electron beam such as evaporation^34^, water radiolysis^35^, wettability modification^36^,^37^ and liquid charging^38^. Droplets were condensed from inside the hydrophobic interstices, although the surface had intrinsic hydrophobicity (Fig. 2). A liquid droplet initiated from inside the hydrophobic cavity grew as the condensation progressed. During this growth procedure, because the grown interface of the water droplet did not penetrate into the other hydrophobic cavities, the morphology of the condensed droplet maintained a near-perfect spherical shape except at the first initiation cavity. From this observation, we supposed that the condensed liquid droplet had partial wetted states, consisting of the wetted part of the first nucleation cavity and the non-wetted part of the other cavities covered by the droplet as it increased in size.

The occurrence of condensation from inside the hydrophobic cavity was described by comparing the grand potentials of non-condensed (*i.e.*, ‘vapor-filled’) and condensed (*i.e.*, ‘liquid-filled’) states in a nanoscale interstice (Fig. 3^39^). The critical gap size can be derived as
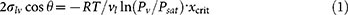
where *x* is the size of gap between nanostructures, *σ* indicates the interfacial force, *θ* denotes the intrinsic contact angle of the plane sample surface, *R* is ideal gas constant and *v_l_* denotes the liquid molar volume and *P* is the pressure of each state. Subscripts *v* and *sat* indicate the vapor and saturated states respectively. According to the relationship described by Eq. (1), the occurrence of condensation from inside the interstice depends on the properties of the condensed fluid, the intrinsic contact angle of the surface, the temperature, and the relative humidity. Therefore, even if the working fluid and cooling surface structure are fixed, the initiation of condensation can be changed by the relative humidity and temperature. In particular, the relative humidity is the most important key factor for condensation. When the relative humidity is less than 1, the right-hand term of Eq. (1) is positive. Therefore, the size criterion for condensation in the interstice is
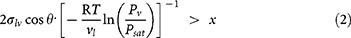
This states that condensation in the interstice can occur when the gap size is less than the length given by the left-hand side of Eq. (2). For *θ* > 90° in air, the left-hand side of Eq. (2) is negative, and thus it is impossible for condensation to occur in the interstice for all length scales. Therefore, only hydrophilic surfaces exposed to air can induce a ‘liquid-filled cavity’ for certain length scales when the relative humidity is less than 1 (Fig. 3b). This phenomenon is known as capillary condensation. However, conventional condensation systems operate under supersaturated conditions, for which *P_v_*/*P_sat_* > 1. For supersaturated conditions, the right-hand side of Eq. (1) becomes negative; therefore,
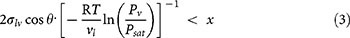
Under these conditions, for a hydrophilic surface the left-hand side term of Eq. (3) is negative, indicating that condensation in the interstice is possible for all length scales. For a hydrophobic surface, the specific length scale that yields condensation from inside the hydrophobic interstice is determined by the type of working fluid, the intrinsic contact angle of the cooling surface, and the condensation conditions (Fig. 3b).

This analysis suggests that the same hydrophobic surface may have completely different wetted states, depending on the surface structure, the type of working fluid, and the condensation conditions. The evaluated critical length from Eq. (3) for condensation in the ESEM facility was ~ 7 nm, based on a saturation pressure evaluated using the temperature of the cooling surface. This length is shorter than the size of the nano-grains of the micro/nano-hydrophobic surface (> 10^1^ nm, Supplementary Section 10). Therefore, the criterion derived by comparing the potential of the non-condensed and condensed states supports the experimentally observed behavior: condensation was initiated from inside the hydrophobic interstice.

Unlike the initiation of condensation, the interface of the water droplet grown from the hydrophobic interstice did not penetrate into the other hydrophobic interstices. To penetrate into the other interstices, the pressure of the liquid droplet must be greater than the vapor pressure across the meniscus formed in the nanostructure. According to the Laplace–Young equation, the pressure difference across the interface is defined by the radius of the interface and the surface tension. The pressure difference increases as the radius decreases. Therefore, for the liquid–vapor interface to penetrate into the interstice, the radius of the liquid droplet must be smaller than the gap size of the interstice. However, this is impossible because the droplets grow from other cavities; the radius of a liquid droplet grown in another cavity must be greater than the gap size of the interstice. Therefore, a liquid droplet that condenses from inside one interstice grows without water penetrating into the other hydrophobic cavities. Consequently, the condensed droplets have partial wetted states.

The partial wetted state of the condensed droplets was confirmed from observations made during coalescence between nucleated droplets. During this process, due to the change in the interface energy, the interface of each droplet was pulled into the volume center of the newly generated droplet by merging. However, because of the fixed interface at the wetted cavities of each droplet, the bases of the droplets did not move in the coalescence direction (Fig. 4a). Therefore, the shape of the merged droplet was deformed from spherical immediately after coalescence. It is well known that spherical Cassie–Baxter droplets easily roll off surfaces, and that Wenzel droplets are highly fixed and stick to surfaces^24^. Thus, these sticky spherical droplets might be located on partially wetted cavities in the surface structure.

The nucleation site density determines the apparent wetted state. If there are numerous nucleation sites, condensed droplets are formed by multiple coalescence of droplets that nucleate inside the structures. Thus, the droplets comprise a large portion of the liquid-filled cavities. Figure 4b shows that the apparent wetted state of a droplet formed by merging many small droplets transformed from an apparent Cassie–Baxter state to an apparent Wenzel state as the amount of the liquid filling the cavity increased. However, if the number of nucleation sites was very small, the condensed droplets remained in an apparent spherical Cassie–Baxter state as they grew and were not fixed, except at the few nucleation cavities.

The apparent change in the wetted state was also found at the conventional condensation conditions. To expand our understanding of conventional condensation, we conducted condensation experiments under atmospheric pressure and visualized the morphologies of the condensed droplets at various heat fluxes using a high-speed camera with an endoscopic lens to obtain a side view (Fig. 5a). The heat flux governs how much steam is condensed by the heat transfer surface; therefore, the heat flux directly changes the number of condensation nucleation sites on the surface. The critical gap sizes for high (~ 440 kW·m^−2^) and low (~ 20 kW·m^−2^) heat fluxes are ~ 0.7 and 4.1 nm, respectively, which are smaller than the scale of the fabricated structures in this study. This implies that the condensed liquid obtained for each condition should wet the hydrophobic nano-interstices. However, spherical Cassie–Baxter droplets were observed on the HMN surface at the low heat flux, while half-spherical Wenzel droplets were observed at the high heat flux. As described above, different nucleation site densities for high and low heat flux conditions cause different portions of the cavities to become filled with condensed liquid, inducing different apparent wetted states of the liquid on the HMN surface. However, because they fundamentally had the same wetted state (the partial wetted state), the apparent Cassie–Baxter condensed droplets under the low-heat-flux condition exhibited sticky characteristic on the vertical condensed surface (Fig. 5b). A spherical droplet (d ~ 1.65 mm) that was shaken by the surrounding perturbations remained at the same position without any roll-off or movement.

The behaviors of the condensed droplets on the HMN surface affect their contact-angle hysteresis, which indicates how strongly a droplet sticks to the surface. Therefore, the contact-angle hysteresis directly influences the departing behavior of the droplet from an HMN surface (Table 1, and Supplementary Information, Section S8). The contact-angle hysteresis was 12.11° on the HS surface and 32.57° on the HMN surface at a high heat flux. The wetted valleys of the hydrophobic structures due to condensed droplets fixed the movement of the droplet interfaces, consequently causing a greater contact-angle hysteresis on the HMN surface. Considering a force balance of a droplet and the surface tension, the maximum size of the droplets on a vertical plate can be represented as follows^40^,^41^,^42^:


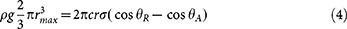
where *c* is a constant. The high contact-angle hysteresis results in a large maximum condensed droplet size in the equation. This corresponds well with the measured maximum radii of the condensed droplets on the smooth and structured surfaces, which were 1.04 and 1.45 mm, respectively. In other words, the high contact-angle hysteresis reduced the removal rate of the condensed liquid droplets, and consequently, the radius of the remaining droplets on the HMN surface increased.

Such sticky behavior of a condensed droplet on the HMN surface is not preferred for practical applications because it deteriorates the self-repelling, anti-sticking, anti-fouling, and self-cleaning characteristics. In particular, these larger condensed droplets provided a higher thermal resistance in the phase-change heat transfer system, reducing the heat transfer performance. This means that micro/nanostructures would fail to induce rapid droplet removal and enhanced heat transfer during condensation. We measured the heat transfer coefficient on the HMN and HS surfaces (Supplementary Information, Section S11). The average heat transfer coefficients of the smooth and micro/nano-structured surfaces were 63.87 and 32.01 kW·m^−2^·K^−1^, respectively. Thus, the heat transfer performance of the structured surface was worse than that of the smooth one, as expected from the theoretical analysis and ESEM observations.

Based on our results, we propose the following to enhance the removal rate of condensed droplets on an HMN surface: ensuring an appropriate gap size between hydrophobic structures for the given operating conditions and controlling the coalescence of the condensed droplets in a certain direction. First, an appropriate gap size or structure morphology for the operating conditions should be considered to achieve high removal rate of condensed droplets. The wetted state of a droplet can be varied during condensation since the condensation is initiated from inside the hydrophobic interstice under supersaturated conditions. If the surface gap size on the HMN surface is less than the criterion for capillary condensation on a hydrophobic surface under S > 1, nucleation occurs on the top of the structures. These droplets can be drained rapidly because they easily roll off the structure when they are in a Cassie–Baxter state. According to Eq. (3), lower saturation conditions have a higher critical size criterion for condensation from a hydrophobic nano-interstice. Therefore, if we cannot realize smaller surface structures, we should regulate the saturation of the condensation system to maintain superhydrophobicity on the HMN surface.

The second proposal concerns controlled coalescence of the condensed droplets. Because a liquid-filled cavity fixes the droplet on the cooling surface, multiple coalescence of condensed drops will induce large portion of liquid filled cavity and it will prevent the rolling and jumping of small water droplets, enhancing the removal rate of condensed droplets on an HMN surface. However, if the number of coalescence is very small, few droplets are fixed at the nucleation cavities. Therefore, the droplets maintain their hydrophobic characteristics by inducing less coalescence on the HMN surface. We can also consider the coalescence behavior. Because coalescence occurs randomly without any preferred direction, the phenomenon cannot influence the condensation in a positive way. However, if the coalescence could be controlled along a certain direction to remove the droplets in that direction, the removal rate of the condensed liquid droplets could be increased significantly as a result of the coalescence interactions, enhancing condensation. Based on these considerations, this work will be extended to improve condensation heat transfer on a superhydrophobic structured surface by using coalescence with an appropriate surface structure gap size or geometry. And, experimental verification for the critical length during condensation environment to support the theoretical criterion will be also followed as future work with robust superhydrophobic structures in several nanometer gaps or cavities.

The present study investigated the mechanism by which superhydrophobic characteristics disappear on an HMN surface as the surrounding conditions change, and proposed guidelines for sustaining the novel properties of an HMN surface based on theoretical and experimental investigations. This induced the loss of the self-cleaning, anti-sticking, and anti-fouling characteristics on the HMN surface under supersaturated conditions. Consequently, a critical gap size for structures was proposed to maintain a superhydrophobic state during condensation. We believe that the postulated criterion could be extended by including nucleation dynamics, and may contribute to better superhydrophobic surface designs by providing a guideline for phase-change, anti-fouling, and self-cleaning systems, as well reducing the frictional pressure drop on a surface.

## Methods

### Sample fabrication

Mechanical polishing was carried out on the 40 × 30-mm^2^ zirconium alloy specimens using 1500# sandpaper to remove foreign substrates and improve the surface finish. Then, the samples were rinsed in acetone, methanol, and deionized (DI) water several times to clean the surface. After drying, the samples were anodized in 0.5 wt.% hydrofluoric acid (HF) solution maintained at 10°C by a thermostat. A 20-V potential was applied for 10 min. After the anodization process, the specimens were rinsed with DI water and dried again. To remove the fluoride remaining on the surface, the specimens were heated to 120°C in a furnace for 100 min. Then, a thin PTFE film was coated on the surface using a spin coater. The sample was held on the spin coater and spun at 500 rpm for 35 s. After that, the sample was heated on a 120°C hot plate for 15 min to remove all solvent.

### ESEM

The behaviors of the condensed droplets were observed using ESEM (FEI Quanta 200 FEG MK). The sample was attached to a stainless-steel sample holder with copper tape and tilted at a 40° angle. A Peltier cooling stage was placed in contact with the stainless steel sample holder, cooling the sample. The cooling temperature was set to ~ 277.25 K and reduced slowly. The vapor pressure in the ESEM chamber was fixed at 890 Pa, and was controlled by a differential pumping system. A beam was injected onto the sample of viewing area of 75.07 × 69.03 *μ*m^2^ with a 10-kV potential through a field emission gun at a working distance of 6.7 mm, which led to the minimal of e-beam effect. Images were captured every 0.5 s using the recoding function of the system.

### Experiment

Condensation experiments were conducted to determine the heat transfer performance and observe the condensate morphologies on the sample surface. Steam at 110°C was generated by boiling distilled water, and supplied to the test chamber containing the sample. The sample was placed in contact with a copper block through which cold water (T ~ 20°C) was circulating to provide cooling so that condensation heat transfer occurred on the sample surface. During the experiment, the temperatures and pressures were measured at steady state. The surface temperature and heat flux were calculated from the measured temperatures assuming one-dimensional (1-D) conduction. Visualization was used to observe the condensation behavior. For a top view, a high-speed camera captured the front face of the sample through a heated transparent window. To observe the side of the sample, an endoscope was inserted into the chamber with an angle of 35°. The detailed experimental setup and procedures are described in the Supplementary Information, Sections S2–5.

## Supplementary Material

Supplementary Information Supplementary Information

Supplementary Information Supplementary Information

Supplementary Information Supplementary Information

Supplementary Information Supplementary Information

Supplementary Information Supplementary Information

Supplementary Information Supplementary Information

## Figures and Tables

**Figure 1:**
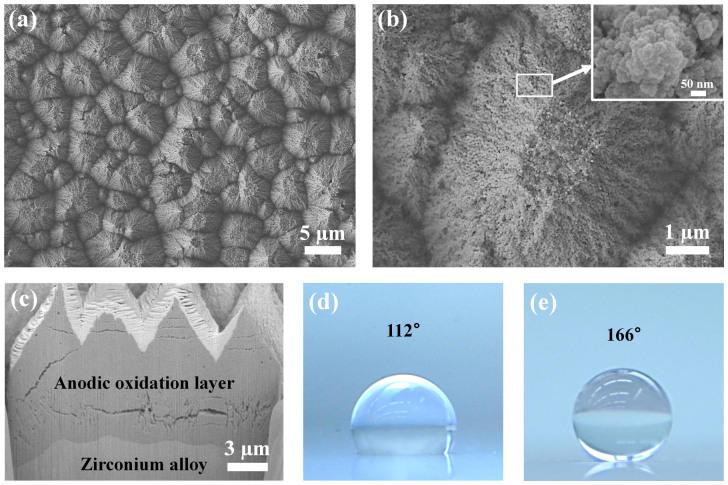
Surface features. (a) FE-SEM images of micro- and nanostructures, (b) at high magnification. (c) SEM image of the FIB-milled cross-section of the surface structures: height ~ 4 μ, spacing between peaks ~ 4.5 μm. A droplet deposited on (d) a hydrophobic surface (θ = 112°), and (e) a superhydrophobic surface (θ = 166°).

**Figure 2:**
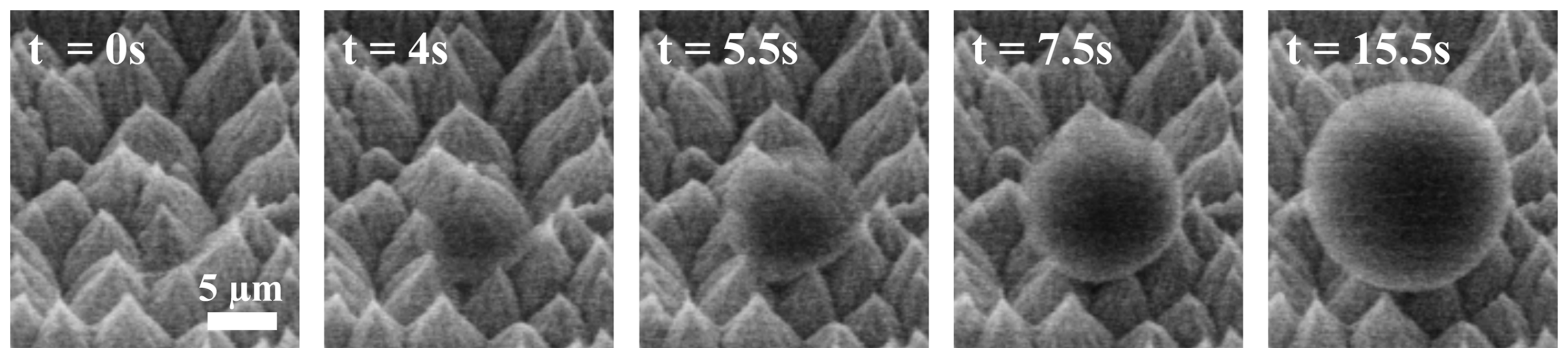
Time-lapse ESEM images of single droplet nucleation (ESEM condition: vapor pressure = 890 Pa, surface temperature = 277.25 K, supersaturation = 1.086, critical length = 7.09 nm).

**Figure 3:**
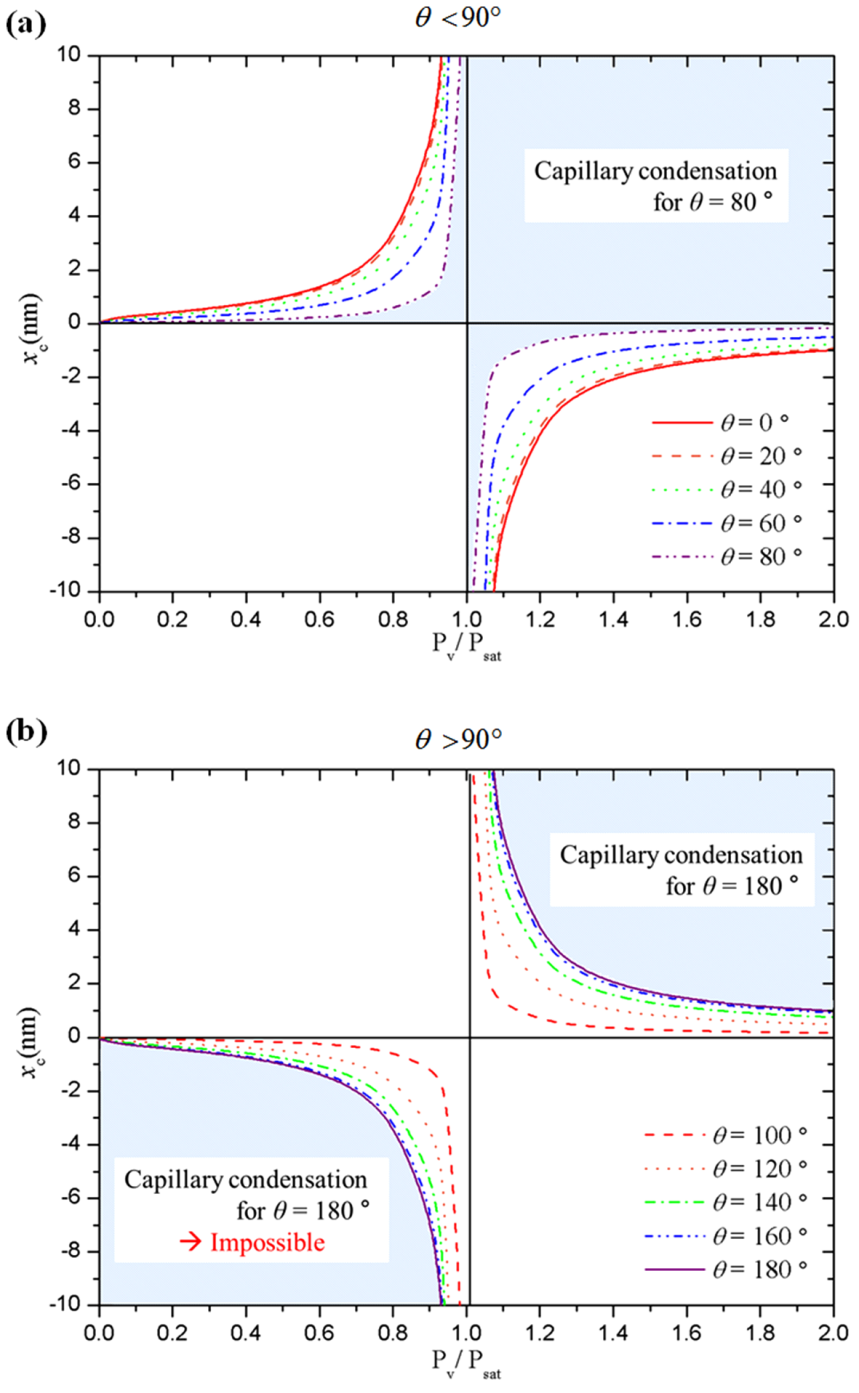
Critical gap size for interstice condensation on (a) hydrophilic and (b) hydrophobic surfaces.

**Figure 4:**
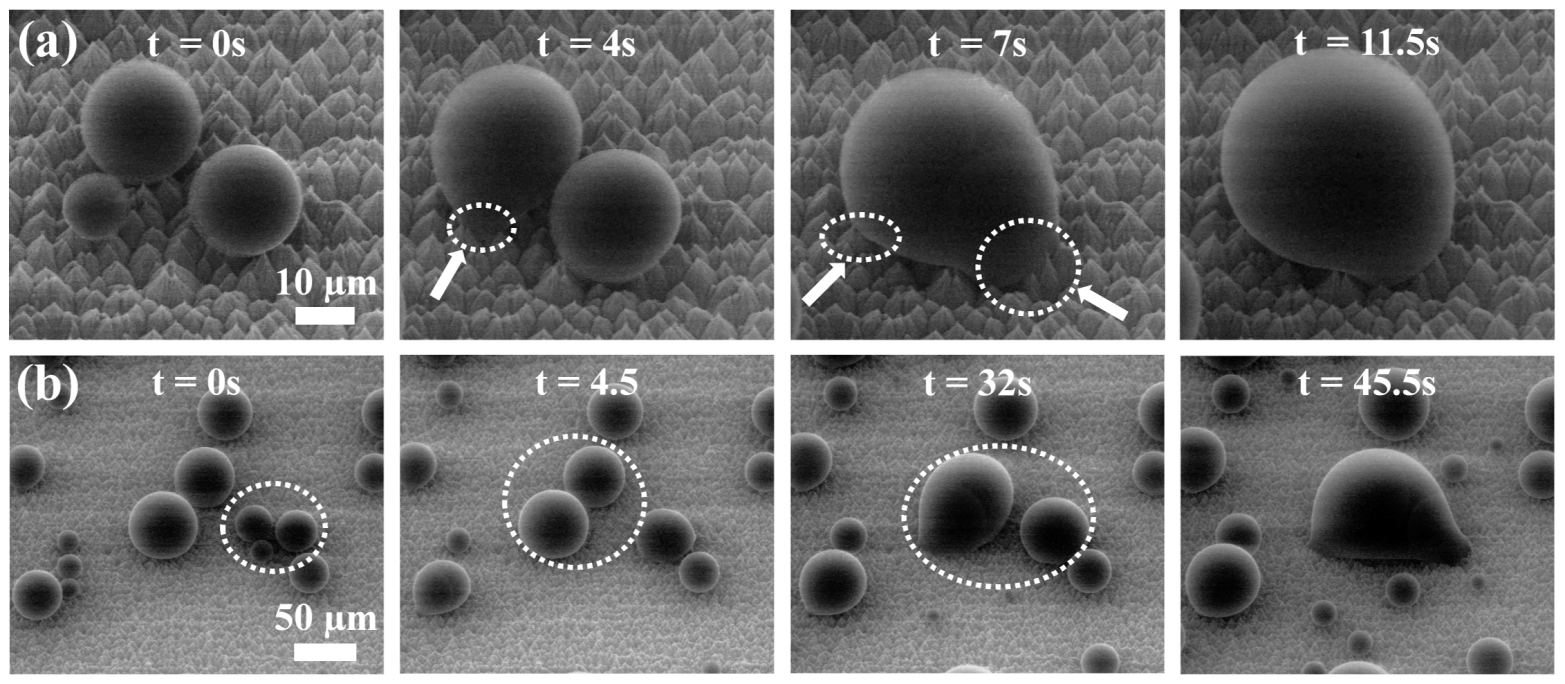
(a) Time-lapse ESEM images of droplet coalescence with a 40° tilt angle. The arrows and dashed lines indicate droplet deformation. (b) Through the coalescence of many droplets, the apparent wetted state of a droplet was transformed from a Cassie–Baxter state (with a small wetted region under the droplet) to a Wenzel state (with a large wetted region under the droplet). The dashed lines indicate the droplets before coalescence.

**Figure 5:**
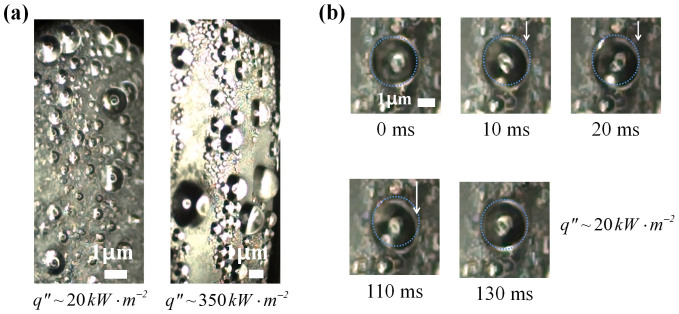
Visualization images (a) tilted side view images obtained from the high-speed camera with an endoscope to observe the droplet morphology at different heat fluxes, (b) sticky spherical droplets.
