# Supplementary material for: Loss of superhydrophobicity of hydrophobic micro/nano structures during condensation
Source: Sci Rep. 2015 Apr 23;5:9901. doi: 10.1038/srep09901 (PMC5386109; doi:10.1038/srep09901)
Supplement: Supplementary Information [file srep09901-s1.pdf]

SUPPLEMENTARY INFORMATION

Loss of superhydrophobicity of hydrophobic micro/nano structures during condensation

HangJin Jo[1,‡], Kyung Won Hwang[2,‡], DongHyun Kim[1], Moriyama Kiyofumi[1], Hyun Sun Park[1], Moo Hwan Kim[1,A,*], Ho Seon Ahn[3,*]

[1]Division of Advanced Nuclear Engineering, POSTECH, Pohang, Gyungbuk, Republic of Korea

[2]Department of Mechanical Engineering, POSTECH, Pohang, Gyungbuk, Republic of Korea

[3]Division of Mechanical System Engineering, Incheon National University, Incheon, Republic of Korea

[‡]These authors equally contributed to this work.

Corresponding Authors: Professors, Moo Hwan Kim (mhkim@postech.ac.kr) and Ho Seon Ahn (hsahn@incheon.ac.kr)

[A] Professor, Moo Hwan Kim is currently working in Korea Institute of Nuclear Safety (KINS) as a president.

S1. ESEM and high-speed videos

Movie S1. Start of condensation on the HMN surface. The movie was made using time-lapse images, which were captured every 0.5 s by ESEM (FEI, Quanta 200). ESEM conditions: vapor pressure = 890 Pa, surface temperature = 277.25 K, supersaturation = 1.0863.

Movie S2. Coalescence of droplets growing by condensation. When a droplet coalesced near another droplet, its shape was deformed, providing evidence of a partial wetted state. The movie was made using time-lapse images, which were captured every 0.5 s by ESEM (FEI, Quanta 200). ESEM conditions: vapor pressure = 890 Pa, surface temperature = 277.25 K, supersaturation = 1.0863.

Movie S3. Transition of the apparent wetted state through the coalescence of many droplets. The movie was made using time-lapse images, which were captured every 0.5 s by ESEM (FEI, Quanta 200). ESEM conditions: vapor pressure = 890 Pa, surface temperature = 277.25 K, supersaturation = 1.0863.

Movie S4. Condensation on the hydrophobic smooth surface captured by the high-speed camera with an endoscope during the experiment. The playback speed is 21×.

Movie S5. Condensation on the hydrophobic micro/nano-structured surface captured by the high-speed camera with an endoscope during the experiment. The playback speed is 21×.

S2. Experimental apparatus

The condensation heat transfer coefficients were experimentally measured using a condensation experimental facility to evaluate the heat transfer performance of the HMN and HB surfaces. The facility consisted of a steam generator, a steam chamber with double windows for visualization, a copper block, and a test section (Fig. S1). The steam generator was controlled with four 4-kW cartridge heaters to generate saturated steam and maintain a constant steam saturation temperature. The temperature and pressure of the saturated vapor was measured using K thermocouples and an absolute pressure transducer, respectively. The working fluid, steam, was passed into the steam chamber through a stainless tube covered by glass fiber for insulation, and was condensed on the zirconium alloy specimens. The test section was cooled by a copper block through which water flowed to transfer the heat energy from the cooled surface. The position of the block was controlled using two supporting jacks. Eight springs were installed to enable full contact with the entire cooling block area under the test section. The steam chamber was heated by cartridge heaters to maintain the saturated temperature and to prevent condensation from forming on the wall of the chamber. The condensed water on the test section was removed by gravity and drained to the atmosphere. The bulk vapor temperature and pressure in the steam

chamber were measured using a thermocouple and an absolute pressure transducer, respectively. The coolant flow rate in the cooling block was measured using a flowmeter, and the inlet and outlet fluid temperatures of the coolant were measured using thermocouples to confirm the heat energy transfer from the test sample to the coolant. A metering needle valve was installed in the outlet line of the steam chamber to accurately adjust the condensate flow rate.

The test section had three ungrounded K thermocouples with diameters of 0.3 mm. Thermal grease was used to reduce the contact resistance between the thermocouples and the test sample. A thermocouple at the center was used to measure the wall temperature of the test section to determine the heat transfer coefficients, while the other two were used to confirm the contact of cooling block with the test section. (Only a thermocouple at the center is shown in Fig. 5(a)) The three thermocouples were installed in holes a distance of 0.405 mm from the condensed surface; the hole diameters were ~0.32 mm. The total size of the test chamber, the size of the cooling area, and the thickness of the test section were 40 × 30 mm, 33 × 23 mm, and 0.7 mm, respectively. Double windows were used to visualize the condensation in the test section (Fig. S2). Indium tin oxide (ITO)-coated glass was used to heat the air between the glass and the polycarbonate to prevent the formation of condensate on the polycarbonate. Because the ITO glass was transparent, visualization with a high-speed camera was possible.

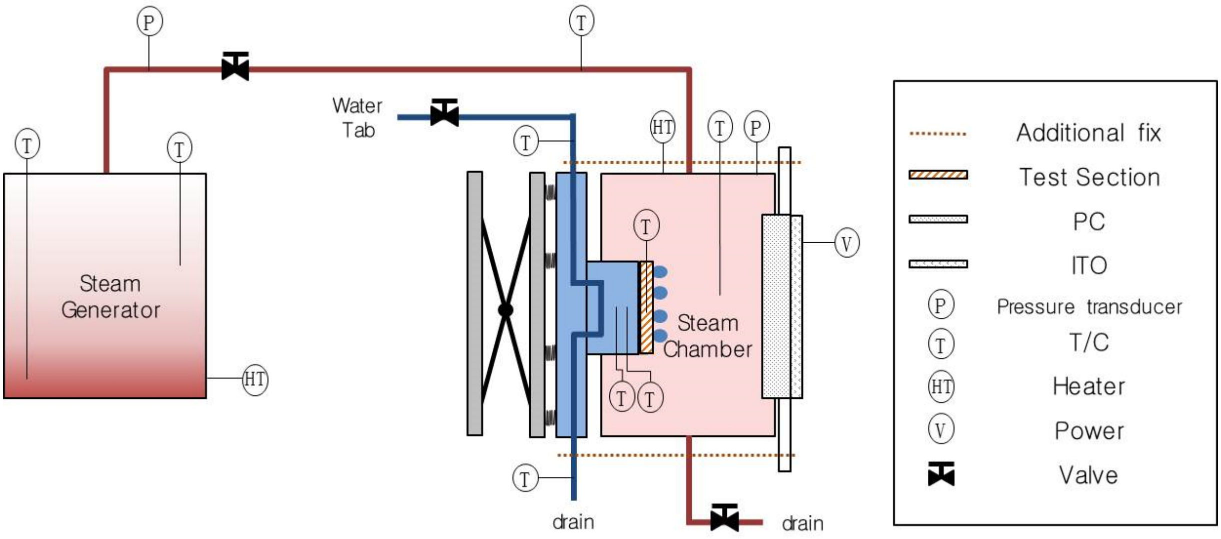

Figure S1. Condensation experimental facility

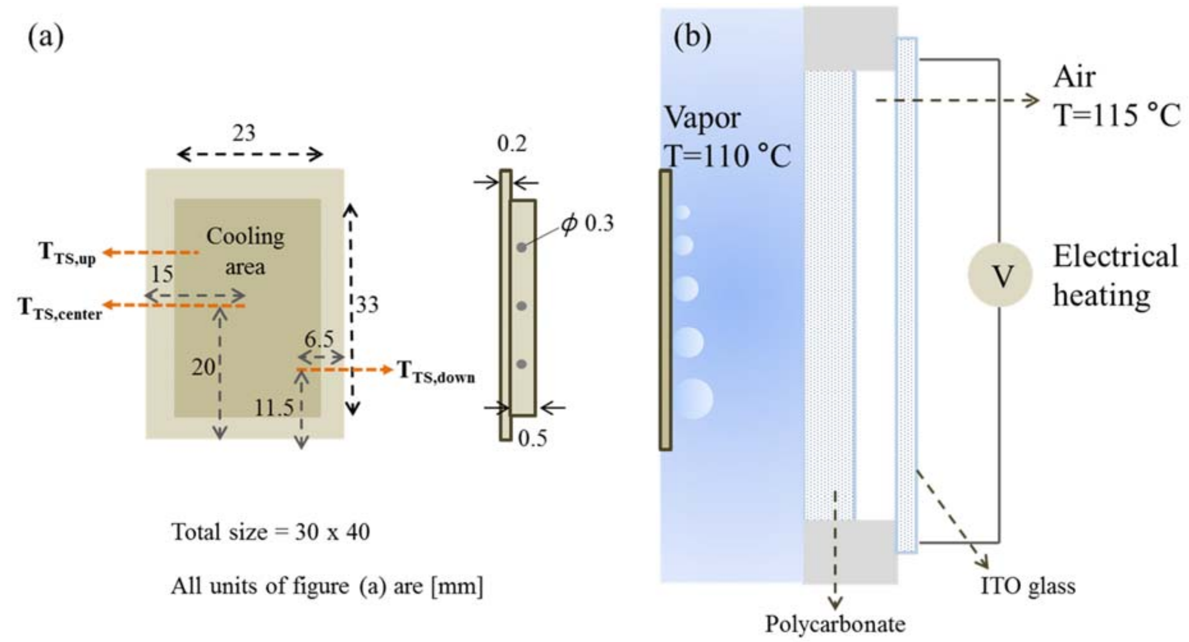

Figure S2. (a) Test section and (b) double window to visualize the condensation.

S3. Experimental procedure

Before the condensation experiments, water in the steam generator was heated at 70°C for 1 h, and a vacuum pump was used to maintain an absolute pressure of 10 kPa in the steam generator and steam chamber to remove non-condensable gases. After the non-condensable gas removal process, the steam generator and chamber were heated to 110°C with the valve between the steam generator and the chamber closed. Once the steam generator temperature reached 110°C, the valve was opened and steam entered the steam chamber. The remaining air in the steam chamber might have accumulated in the lower half of the chamber because the density of air (1.28 kg/m^3) is higher than that of steam (0.81 kg/m^3) at the measured temperature of 109.3°C and pressure of 140.0 kPa. Therefore, the air under the steam layer was removed through a drain line with a fully open valve over a period of 2 min, during which the flow rate control valve was opened and closed fifteen times. Thus, the remaining non-condensable gas was expected to be entirely exhausted to the atmosphere because the gas and condensate mixtures were continuously drained during the entire experiment process. Tap water flowed through the cooling block at 6.5 L/min for the high-heat-flux case and at 3.0 L/min for the low-heat-flux case. The copper block was attached under the test section to cool it down. The position of the block was adjusted using the two supporting jacks. Thermal grease was placed on a contact surface between the block and the test sample as a lubricant. The contact between the cooling block and the test

section during condensation was confirmed by temperature measurements in the test section. Each condensation experiment typically lasted 1 hr.

S4. Data measurement and calculations

The main measured parameters were the heat transfer coefficient and the heat flux from the condensed surface to the coolant. The temperature difference in the cooling block was measured with two K thermocouples, 0.5 mm in diameter and located a distance of 7 mm parallel to the direction of the heat energy transfer from the vapor to the coolant. The heat flux was estimated from the temperature difference assuming 1-D conduction at the cooling block. The condensed surface temperature was also required for the heat transfer coefficient; it was calculated from the measured heat flux and wall temperature beneath the condensed surface, assuming 1-D conduction. The wall temperature was measured using a K thermocouple, 0.3 mm in diameter and located a distance of 0.405 mm from the condensed surface.

The heat flux was calculated from the temperature difference in the cooling block using the heat diffusion equation, assuming 1-D conduction and steady-state conditions:

$$q'' = -k\frac{dT}{dz} = k\frac{\Delta T}{z} \quad (1)$$

where is k, z, and ΔT are the thermal conductivity (kW/m/K) of the cooling block, the distance (0.007 m) between the thermocouples, and the temperature difference between the thermocouples (T_1 and T_2). $T_{surface}$ was estimated from equation (1) based on the measured temperature $T_{TS,\,center}$ at the center of the test sample and the heat flux. The distance from the condensing surface to center of the thermocouple was measured from the camera images. (z_{TS} = 0.40500 mm ± 0.00366 mm). The heat transfer coefficient was calculated using Newton's cooling law:

$$q'' = h\left(T_{sat} - T_{surface}\right) \quad (2)$$

where q'', h, T_{sat}, and $T_{surface}$ are the heat flux (kW/m^2), heat transfer coefficient (kW/m^2/K), temperature (K) of the saturated vapor, and temperature of the condensed surface, respectively.

S5. Uncertainty analysis

The uncertainty of the heat flux can be written as follows:

$$\frac{U_{q''}}{q''} = \sqrt{\left(\frac{U_k}{k}\right)^2 + \left(\frac{U_z}{z}\right)^2 + \left(\frac{U_{T_1}}{T_1 - T_2}\right)^2 + \left(\frac{U_{T_2}}{T_1 - T_2}\right)^2} \quad (3)$$

where $U_{q''}$, U_k, U_z, U_{T1}, and U_{T2} are the uncertainties of the heat flux (kW/m^2), thermal conductivity (kW/m/K), distance (m) between the thermocouples for the heat flux measurement, T_1 (K), and T_2 (K), respectively. The uncertainty of the wall temperature is

$$U_{T_{surface}} = \sqrt{U_{T_{TS,center}}^2 + \left(\frac{q''}{k} U_z\right)^2 + \left(\frac{q''z}{k^2} U_k\right)^2 + \left(\frac{z}{k} U_{q''}\right)^2} \quad (4)$$

The temperatures were measured using K thermocouples, which were carefully calibrated using a reference resistance temperature detector. The uncertainty of the temperature measurements was assumed to be ±0.2 K. The uncertainty of the thermal conductivity was assumed to be 2%. The estimated maximum uncertainties of the heat flux and the wall temperature of the test sample were 19.7 kW/m^2 and 0.69 K.

S6. Smooth hydrophobic surface

To verify the suitability of the experimental system and the procedure for measuring condensation heat transfer, the heat fluxes and the vapor–surface temperature differences were measured on the PTFE-coated smooth zirconium alloy surfaces at a saturated pressure of 140 kPa, as shown in Fig. S3, and the results were compared to the Hanneman and Mikic[1] correlation, which takes the thermal conductivity of the condensed surface and the departing droplet diameter effects into account. The average heat transfer coefficient on the PTFE-coated smooth surfaces was 63.9 kW/m^2/K. The coefficient predicted by the correlation was 79.8 kW/m^2/K at the same conditions. When taking into account the deviation of the departing droplet diameter, the predicted heat-transfer coefficients ranged from 72.9 to 88.0 kW/m^2/K, as shown by the error lines in the figure. The difference between the experimentally measured average heat transfer coefficients and the predicted values with the larger maximum diameter (r_{max} = 1.19 mm) was 14.1%. Considering the uncertainty of the temperature differences between the vapor and the condensing wall, the present experimental results for dropwise condensation on PTFE-coated smooth surfaces were in good agreement (within 14.1%, on average) with the Hanneman and Mikic correlation.

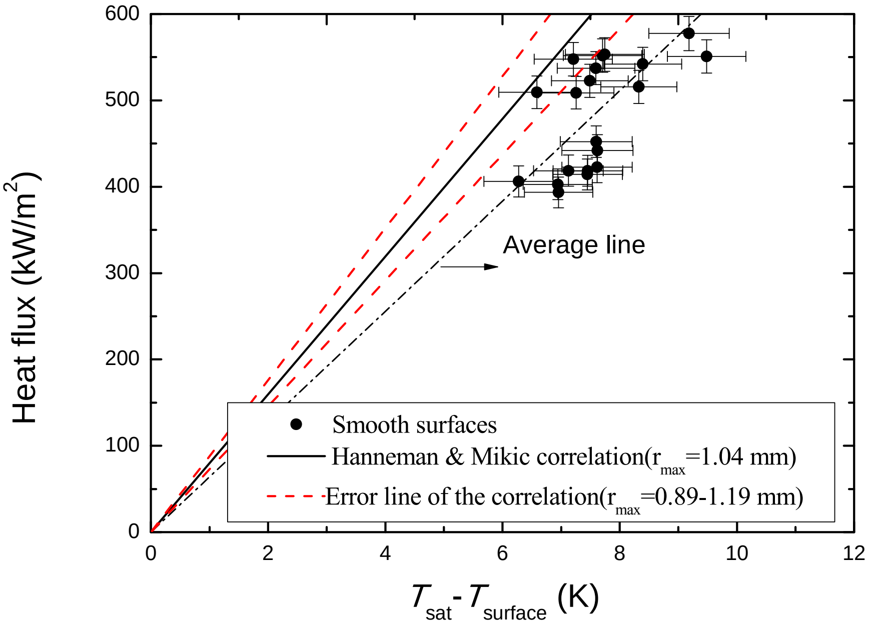

Figure S3. Heat transfer measurements for the PTFE-coated smooth surfaces

S7. PTFE coating and measuring its thickness

A special PTFE material (AF1600, DuPont Polymers, Inc.) was used for the hydrophobic coating on the HS and HMN surfaces. The AF1600 was mixed with FC40 solvent (3M Inc.) to enhance the coating process, and spin-coated onto the samples.

The thickness of the PTFE coating layer was measured using a surface profiler (Veeco Inc., Dektak 150) offering 10-nm thin film resolution. However, accurate values could not be obtained because the roughness of the zirconium surfaces was greater than the film thickness. Thus, PTFE was coated onto silicon oxide surfaces with an average roughness of 1.75 nm using the same method. After the coating process, the measured thickness obtained from the surface profiler was 94 nm. The estimated temperature drop due to the thickness of the PTFE film was 0.2 K at a heat flux of 500 kW/m^2. Thus, the thermal resistance of the coating layer was neglected in this study.

S8. Measurement of contact angle hysteresis and maximum droplet radius

Top view images of the surface were captured by the high-speed camera through the double windows during condensation. The condensed droplet radii just before the droplets departed from the surface (*i.e.*, maximum radii) were measured from these captured images using

ImageProWin4 (Fig. S4(c)). Instead of using a double window, the endoscope was used to observe the contact angle hysteresis of the condensed droplets on the surface. As shown in Fig. S4(a), the endoscope was tilted 35° from the condensed surface and inserted into the steam chamber to capture the side view of the surface. The contact angle hysteresis was also determined using ImageProWin4 immediately before the droplet departed (Fig. S4(d)).

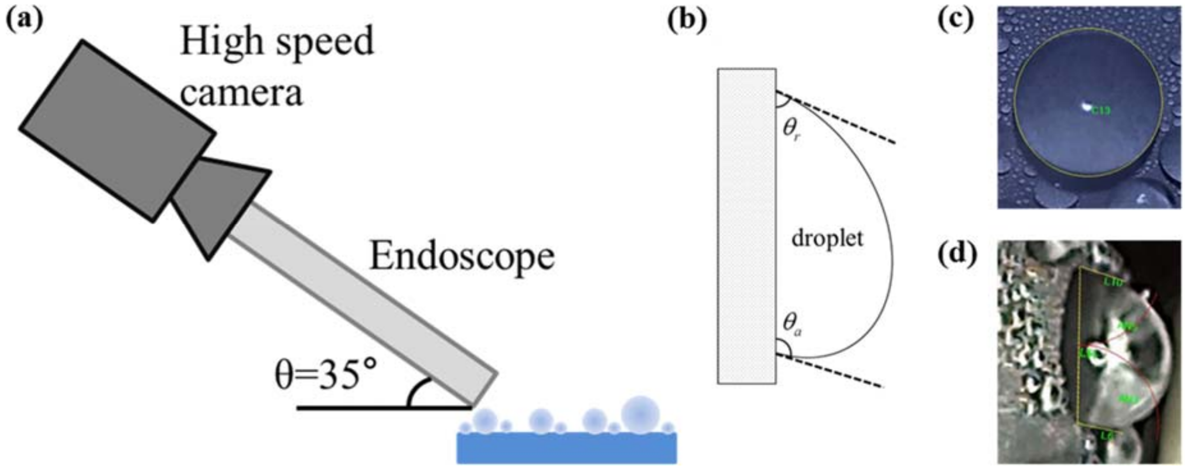

Figure S4. (a) Schematic diagram of an endoscope installation to obtain a side view of the condensation. (b) Schematic diagram of contact angle hysteresis. (c) Maximum radii of the condensed droplet obtained using the visualized images. (d) Measurement of the contact angle hysteresis.

S9. Uniformity of the PTFE layer on the micro/nanostructures

The uniformity of the PTFE coating on the structured surfaces was characterized by high-resolution FE-SEM and by analyzing the element components of the PTFE-coated surface with energy-dispersive x-ray spectroscopy (EDS). EDS is an analytical technique used to provide an elemental analysis of a sample. In Fig. S5(a) and (b), no differences could be found between without and with PTFE-coated ones from the SEM images. The structures could be clearly seen from the images. Thus, there was no aggregation of the PTFE on the surfaces, and the PTFE was spread evenly. The PTFE-coating layer on the structures was characterized by an elemental analysis using EDS, which quantitatively measured the atomic proportion of each element (Fig. S5(d)). The peaks indicated the existence of each element, such as zirconium, fluorine, oxygen, and platinum, on the HMN surface within the area marked in Fig. S5(c). Zirconium and oxygen were present in the structures on the surface. Platinum was present in the coating applied during the technical process for SEM and EDS, and fluorine was present in the PTFE layer. The atomic

proportions of fluorine on the HMN and HS surfaces are listed in Table S1. The amount of fluorine did not differ between the HMN and HS surfaces. Thus, the PTFE was spread evenly on the structures, forming a uniform coating on the HS surfaces.

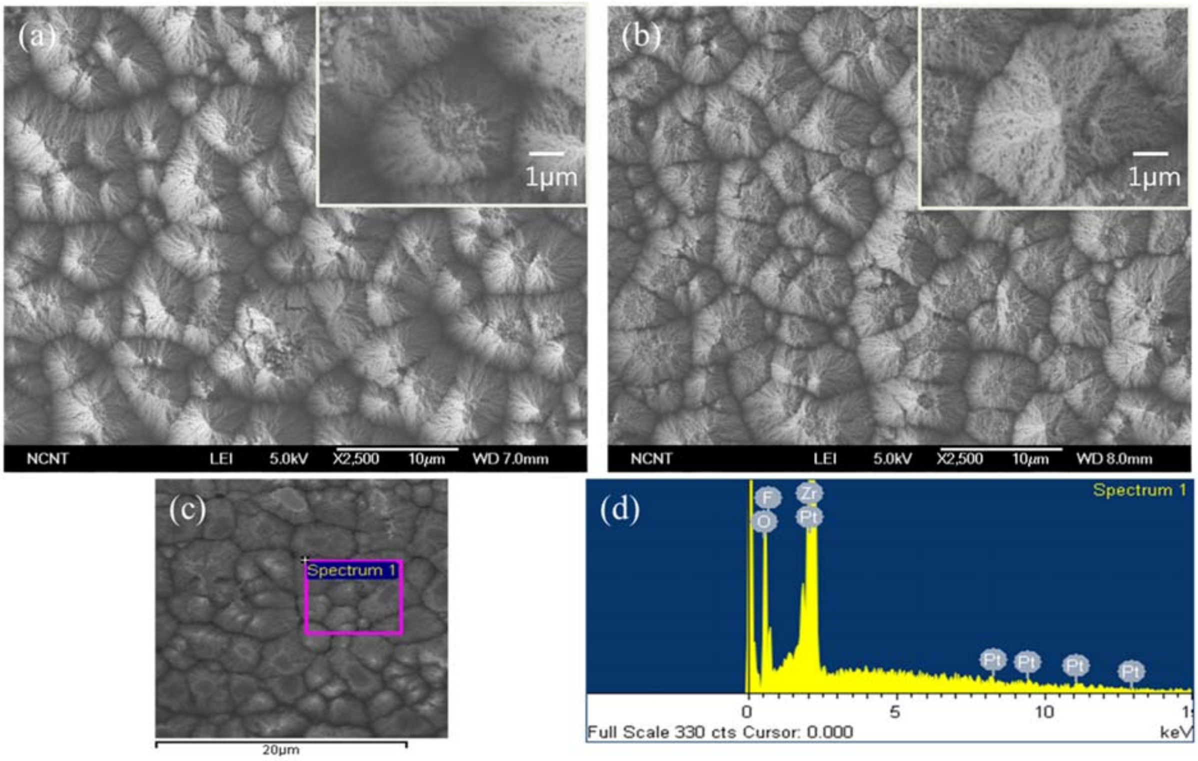

Figure S5. Uniformity of the PTEF coating on the surface at a few tens micro scale: (a) SEM image of a bare structured surface (inset: at high magnification), (b) SEM image of the PTFE-coated structured surface (inset: at high magnification), (c) SEM image of the HMN surface for the EDS analysis area (marked area) and (d) EDS spectrum of the HMN surface showing peaks characterizing each element on the specimen surface.

Table S1. Atomic proportion of fluorine on the HMN and HS surfaces.

	HMN surface	HS surface
Fluorine atomic proportion (%)	15.67	13.92

The coating layer of PTFE on nanostructures was also characterized by analyzing the element components of the PTFE-coated surface using EDS at nanoscale (Fig. S6). Atomic proportion of

fluorine on the structures at nanoscale is 21.84 % which is higher than microscale. The difference of fluorine proportion between micro-scale and nanoscale is enough to be neglected. EDS result confirms uniform Fluorine (PTFE) layer on nano-grains of the surface within about 200 nm X 150 nm size like micro-scale EDS results. SEM image reveals PTFE coated micro/nanostructures maintain its geometrical characteristics like before the coating process (Fig. S5 (a) and (b)). And, even though there is PTFE coating layer on the nanostructures, the characteristic length of the modified surface will not change as shown in Fig. S 7(e). Thus, PTFE is expected to be coated on nanostructures which maintain its original structure after its coating.

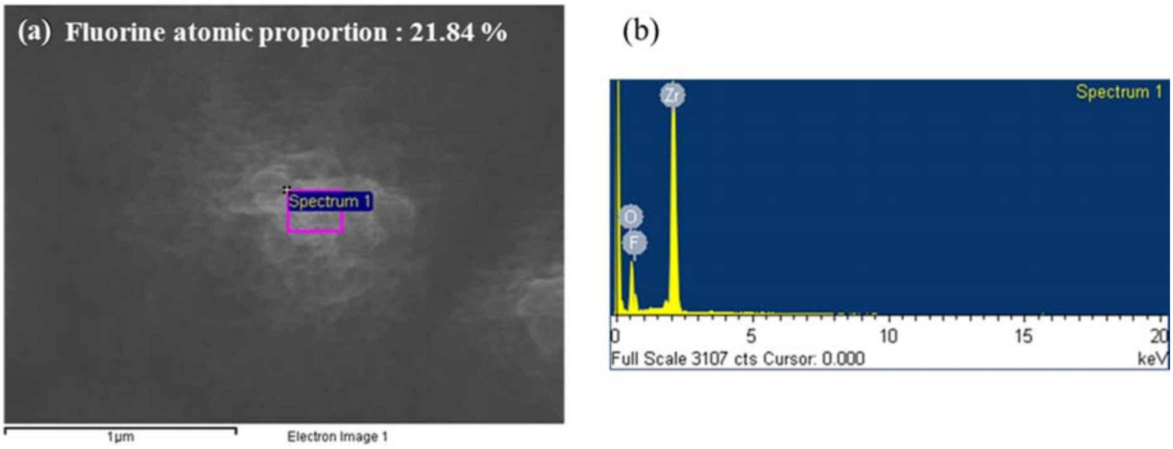

Figure S6. Uniformity of the PTEF coating on the surface at a few hundreds nanoscale: (a) SEM image of the nanostructures and the area marked by box is characterized by EDS and (b) EDS spectrum of the HMN surface at nanoscale showing peaks characterizing each element on the specimen surface.

S10. Geometrical characteristic of the hydrophobic micro/nanostructured surface

In this study, the hydrophobic micro/nanostructured surface was obtained by an anodic oxidation method. The fabricated surface geometry was characterized using high-resolution FE-SEM with FIB. The surface has the nano-grains of several tens of nanometer with micro-groove. The height of micro-groove structures was ranged from 4.3 to 6.4 μm, and the distances between the tops of micro-groove were 3.6 ~ 5 μm. The nano-grains were formed on the micro-groove structures. The size of the nano-grains of the micro/nano-hydrophobic surface was measured from SEM images using image processing program ‘Image Pro Win 4’. The size of the nano-grains was ranged from 22 to 49 nm (one pixel of images was 2 nm.). PTFE coating on the micro/nanostructured surface was confirmed in S9. So, the hydrophobic micro/nanostructured surface could be regarded as the geometrical model as shown in Fig. S7. Based on that, we compared the diameter of the nano-grains and the evaluated critical length from Eq. (3) for

condensation to determine the occurrence of the condensation from inside the hydrophobic interstice.

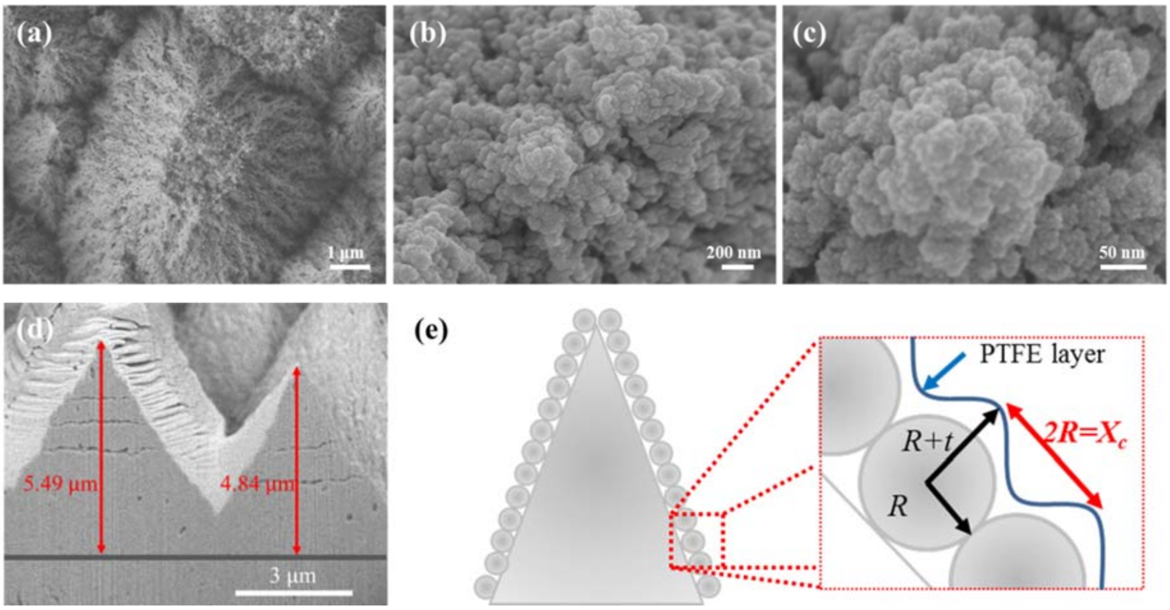

Figure S7. (a), (b), (c) SEM images of the hydrophobic micro/nanostructured surface, (d) with FIB and (e) the geometrical model for the hydrophobic structured surface.

S11. Heat transfer performance on the PTFE coated the micro/nanostructured surfaces

The heat flux and the surface temperature results of the condensed surface and bulk vapor were displayed in Fig. S8. The slope of these data is a heat transfer coefficient which shows the efficiency of the heat transfer. The average heat transfer coefficients of smooth surfaces and PTFE coated micro/nanostructured surfaces were 63.87 kW/m^2/K and 32.01 kW/m^2/K, respectively. The heat transfer performances of the structured surfaces reduced than smooth ones. This result was corresponding to grand potential analysis and ESEM characterization.

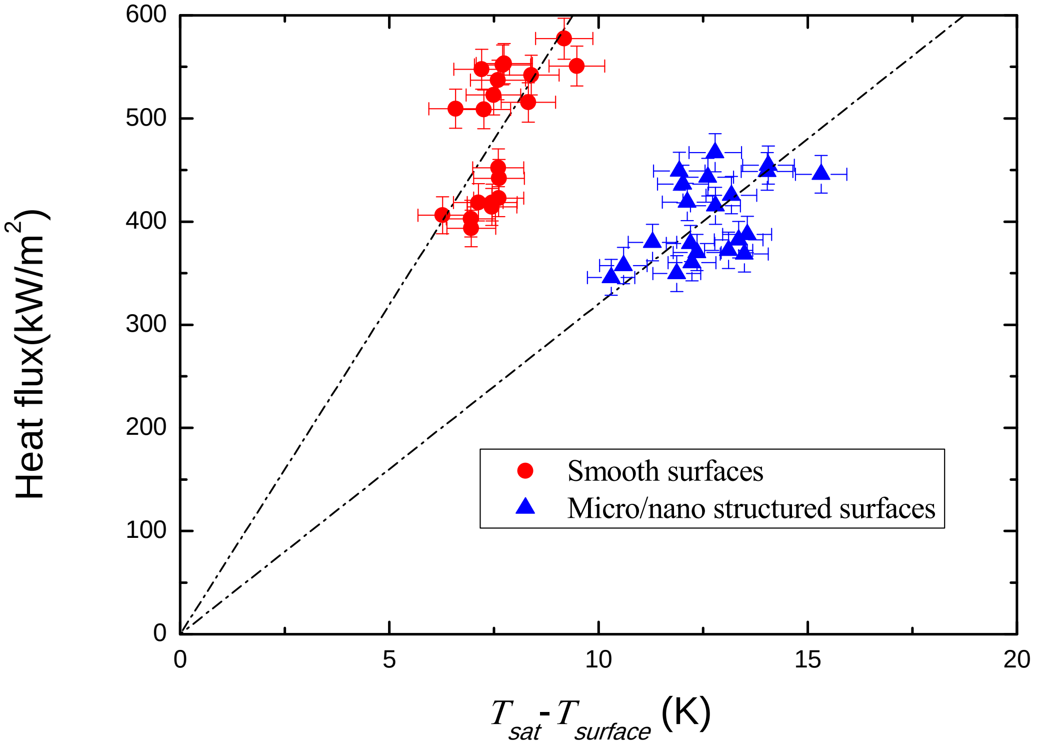

Figure S8. Heat transfer measurements for the PTFE-coated smooth surfaces and PTFE coated Micro/nano structured surfaces.

REFERENCE

1. Hannemann, R. & Mikic, B. An analysis of the effect of surface thermal conductivity on the rate of heat transfer in dropwise condensation. *International Journal of Heat and Mass Transfer* 19, 1299-1307 (1976).
